# Phosphoglycerate Mutase 1 Predicts the Poor Prognosis of Oral Squamous Cell Carcinoma and is Associated with Cell Migration - Erratum

**DOI:** 10.7150/jca.42410

**Published:** 2020-01-24

**Authors:** Dadong Zhang, Heming Wu, Xiaomin Zhang, Xu Ding, Min Huang, Meiyu Geng, Hongwei Li, Zuoquan Xie

**Affiliations:** 1 Division of Anti-tumor Pharmacology, State Key Laboratory of Drug Research, Shanghai Institute of Materia Medica, Chinese Academy of Sciences, Shanghai 201203, China.; 2 Jiangsu Key Laboratory of Oral Diseases, Nanjing Medical University; Department of oral and maxillofacial surgery, Affiliated Hospital of Stomatology, Nanjing Medical University, Nanjing 210029, China.; 3 University of Chinese Academy of Sciences, Beijing 100049, China.

In our paper 1, the Figure 2C, Figure 2E and Figure 4B should be corrected as the following Figure A1, Figure A2 and Figure A3.

## Figures and Tables

**Figure A1:**
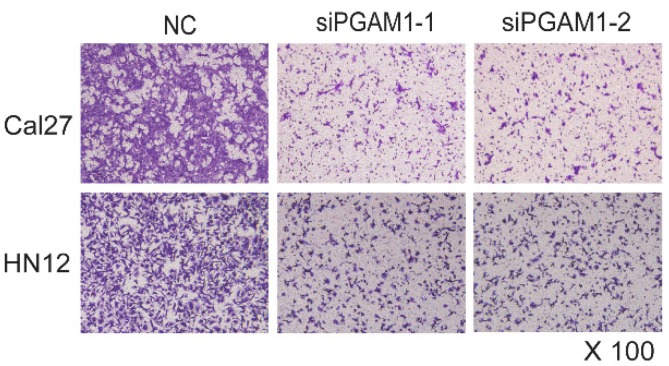
(Figure 2C). **Decreased cell migration after knocking down PGAM1.** Cell migration (24 h) was dramatically decreased after knocking down PGAM1 in both Cal27 and HN12 cells.

**Figure A2:**
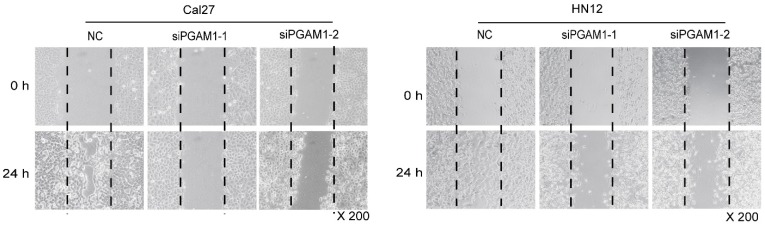
(Figure 2E). **Decreased cell mobility after knocking down PGAM1**. Cell mobility was decreased after knocking down of PGAM1 in both Cal27 and HN12, as determined by the wound-healing assay.

**Figure A3:**
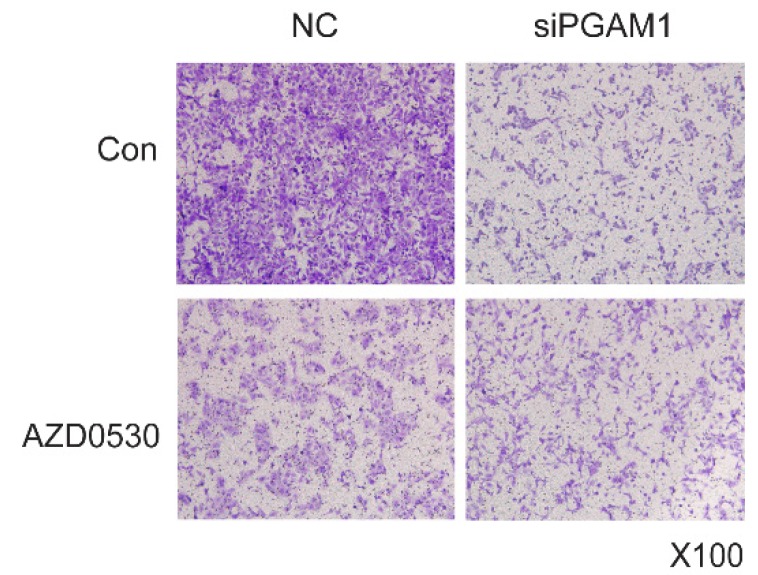
(Figure 4B). **SRC phosphorylation is required for PGAM1-mediated cell migration.** Cell migration was decreased by AZD0530 or PGAM1 knock down, while pretreatment with AZD0530 abolished a further decrease in cell migration when PGAM1 was knocked down.
